# Nitrosative Stress in Autism: Supportive Evidence and Implications for Mitochondrial Dysfunction

**DOI:** 10.1002/advs.202304439

**Published:** 2024-02-21

**Authors:** Richard E. Frye, Shannon Rose, Irena Voinsky, David Gurwitz

**Affiliations:** ^1^ Autism Discovery and Treatment Foundation Southwest Autism Research and Resource Center Rossignol Medical Center Phoenix AZ 85006 USA; ^2^ Arkansas Children's Research Institute and Department of Pediatrics University of Arkansas for Medical Sciences Little Rock AR 72202 USA; ^3^ Department of Human Molecular Genetics and Biochemistry Faculty of Medical and Health Sciences Tel Aviv University Tel Aviv 69968 Israel; ^4^ Sagol School of Neuroscience Tel Aviv University Tel Aviv 69978 Israel

**Keywords:** autism spectrum disorder (ASD), metallothionein, mitochondrial dysfunction, *MT2A*, nitric oxide (NO), NO synthase (NOS), tetrahydrobiopterin

## Abstract

A recent study by the Amal team published in this journal in May 2023 proved for the first time the link of nitric oxide (NO) with autism spectrum disorder (ASD), thereby opening new venues for the potential use of neuronal nitric oxide synthase (nNOS) inhibitors as therapeutics for improving the neurological and behavioral symptoms of ASD. The authors conclude that their findings demonstrate that NO plays a significant role in ASD. Indeed, earlier studies support elevated NO and its metabolites, nitrite, and peroxynitrite, in individuals diagnosed with ASD. Dysregulated NOS activity may underlie the well‐documented mitochondrial dysfunction in a subset of individuals with ASD. Strategies for treating ASD shall also consider NO effects on mitochondrial respiration in modulating NOS activity. Further experimental evidence and controlled clinical trials with NOS modifiers are required for assessing their therapeutic potential for individuals with ASD.

A recent study by the Amal team published in this journal in May 2023^[^ ^1^ ^]^ proved for the first time the link of nitric oxide (NO) with autism spectrum disorder (ASD), thereby opening new venues for the potential use of neuronal nitric oxide synthase (nNOS) inhibitors as therapeutics for improving the neurological and behavioral symptoms of ASD. Notably, an earlier study from Prof. Amal and his team applied S‐nitrosylation (SNO) proteomics analysis of cortical tissues of the Shank3 mouse ASD model and reported elevated S‐nitrosylation of mitochondrial proteins required for ATP production and the electron transfer chain.^[^ ^2^ ^]^ Indeed, S‐nitrosylation was already reported over 30 years ago as a key product of the NO pathway,^[^ ^3^ ^]^ and dysregulated neurotransmitter‐receptor interaction following protein nitrosylation was already reported in 1985.^[^ ^4^ ^]^ Obviously, the potential use of nNOS inhibitors as ASD therapeutics must first be tested in clinical trials, which to our knowledge are not yet being done (according to a clinicaltrials.gov search of June 25, 2023). A large part of the study by the Amal team^[^ ^1^ ^]^ reports observations in the mutated Shank3 and Cntnap2 ASD mouse models with nNOS inhibitors as well as WT mice with an NO donor. Further, in vitro findings in iPSC‐derived neurons from ASD individuals with SHANK3 mutations. These human iPSC cells were treated with NO inhibitors. They also used human‐based cell line with SHAKNK3 and nNOS double knock down to prove the mechanism. They further used state‐of‐the‐art SNOTRAP technology to identify SNO‐proteins and 3‐nitrotyrosine in blood samples, which are part of the nitrosative stress hypothesis. The clinical measurement of NO by the Amal team^[^ ^1^ ^]^ was done on blood samples to measure nitrotyrosine and SNO proteomics. They measured 3‐nitrotyrosine, which is an NO‐biomarker for nitrosative stress, and conducted state‐of‐the‐art SNOTRAP‐based mass spectrometry technology combined with systems biology analysis to identify the SNO‐proteome. Additionally, they showed biological processes and pathways which were enriched following of S‐nitrosylation in the blood samples of children with ASD. Here, we provide further direct and indirect support for the conclusions of the Amal lab work from earlier measurement studies with ASD and control human samples, as well as other safe and effective available therapeutics to modulate NO production in ASD.

Higher levels of NO were previously reported in ASD by Zoroğlu et al. in 2003^[^ ^5^ ^]^: this study found elevated plasma nitrite levels, a metabolite of NO, in 26 children with ASD compared with 22 neurotypical children (FD = 1.38; p < 0.001). Another study from the same team found higher levels of NO in erythrocytes from 27 children with ASD compared with 30 matched controls (FD = 1.47; p < 0.0001).^[^ ^6^ ^]^ Elevated NO in plasma samples from children with ASD was also reported by others.^[^ ^7^ ^]^ Additionally, higher levels of the NO metabolite nitrite were reported in saliva from 126 ASD children compared with 129 neurotypical children (FD = 1.87; p < 0.0001).^[^ ^8^ ^]^

The James laboratory at Arkansas Children's Research Institute demonstrated nitrosative stress (as indexed by 3‐Nitrotyrosine) in ASD plasma,^[^ ^9^ ^]^ post‐mortem brain tissue^[^ ^10^ ^]^ and lymphoblastoid cell lines (LCLs)^[^ ^11^ ^]^ as early as 2012. Nitrosative stress markers were associated with a decreased (unfavorable) glutathione redox ratio which the James laboratory previously demonstrated to be a key metabolic abnormality associated with ASD and impaired methylation capacity in 2004.^[^ ^12^ ^]^ Furthermore, that index of nitrosative stress was found to predict adaptive behavior in children using partial least squares analysis when combined with other metabolic biomarkers of oxidative stress in a general group of children with ASD^[^ ^13^ ^]^ and by itself in children with ASD and mitochondrial disease.^[^ ^14^ ^]^

Direct and indirect support for elevated NO in ASD comes from observations on mitochondrial dysfunction^[^ ^15^, ^16^, ^17^ ^]^ and altered mitochondrial morphology^[^ ^18^ ^]^ in ASD. In 2009, the James laboratory demonstrated that exposing lymphoblastoid cells to NO decreased the mitochondrial membrane potential to a greater extent in the ASD cell lines as compared to the control cell lines.^[^ ^19^ ^]^ NO is known to damage mitochondrial respiration in neurodegenerative diseases.^[^ ^20^, ^21^ ^]^ This was clearly demonstrated for noradrenergic locus coeruleus neurons.^[^ ^22^ ^]^

Perhaps most important, is the known mechanism for elevated nitrosative stress in ASD and its treatments. Nitric oxide synthase (NOS) uses tetrahydrobiopterin (BH_4_) as a non‐degradable cofactor to produce NO (**Figure** **1**). In the absence of BH_4_, NOS becomes uncoupled, and the product of NOS is peroxynitrite (Figure 1). Low cerebrospinal fluid concentrations of BH_4_ have been found in some children with ASD below the age of 7 years due to a deficit in either BH_4_ production or recycling.^[^ ^23^ ^]^ BH_4_ supplementation has been shown to have positive effects on ASD behavior in early open‐label studies and controlled clinical trials at low doses (1–6 mg kg^−1^)^[^ ^23^ ^]^ and at higher doses (20 mg kg^−1^) in a double‐blind placebo‐controlled trial^[^ ^24^ ^]^ and an open‐label biomarker trial.^[^ ^25^ ^]^ Interestingly, in the biomarker trial, response to BH_4_ treatment was related to arginine and citrulline, implicating NOS activity, and to the reduced to oxidized pterin ratio, suggesting that the redox state of the patient was critically important.^[^ ^25^ ^]^ These findings led to the notion that low levels of BH_4_, either through reduced production, increased consumption, or oxidation from an unfavorable redox environment, resulted in the uncoupling of NOS and the production of peroxynitrite rather than nitric oxide. This, in turn, creates an unfavorable redox environment, which further oxidizes BH_4_, thus creating a vicious spiral of low BH_4_. Thus, inhibiting NOS in this context, as done in the Amal team study^[^ ^1^ ^]^ would be beneficial. Supplementing BH_4_ could be beneficial in some ASD patients, as shown in previous studies. For the non‐responders who appear to have unfavorable redox balance, treatment to improve redox balance may be beneficial as an add‐on to BH_4_ (Figure 1).

**Figure 1:**
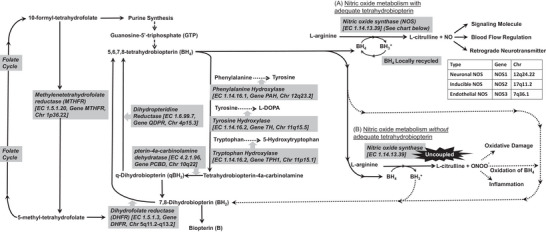
The role of tetrahydrobiopterin (BH_4_) in metabolism. Tetrahydrobiopterin (BH_4_) is essential for phenylalanine metabolism and the production of tyrosine, monoamine neurotransmitters (dopamine, norepinephrine, epinephrine, serotonin, and melatonin) and nitric oxide. BH_4_ is produced de novo from the purine guanosine and can be recycled through folate‐dependent and non‐dependent pathways. The availability of BH_4_ at adequate concentrations is particularly important for nitric oxide (NO) metabolism. A) When BH_4_ concentrations are adequate, NO is produced. In the normal nitric oxide synthase (NOS) reaction, BH_4_ is not used up and is recycled locally. B) However, when BH_4_ is not provided in adequate concentrations NOS becomes uncoupled and a peroxynitrite (ONOO^−^) is produced instead of NO. Peroxynitrites are highly reactive molecules that can cause oxidative damage and inflammation and oxidize BH_4_ into BH_2_, resulting in BH_4_ depletion. In addition, when NOS is uncoupled, BH_4_ is reduced into BH_2_ instead of being recycled, thereby further depleting BH_4_. Gray boxes outline enzymes responsible for the reactions with their gene name and abbreviation, chromosomal location, and enzyme commission (EC) number. Solid lines depict normal metabolic pathways while dashed lines depict metabolic pathways when BH_4_ is in low supply. There are three different types of NOS which are outlined in the chart. Adapted from^[^ ^25^ ^]^ with permission per Creative Commons Attribution‐NonCommercial‐No Derivative Works 3.0 Unported License.

Notably, in our recent RNA‐sequencing study of whole blood samples from 73 Israeli and American children with ASD compared with 26 matched neurotypically developing children, we observed reduced expression of *MT2A*, a gene coding for metallothionein 2A (FD = 0.72; p = 0.0057).^[^ ^26^ ^]^ Metallothioneins play a key role in the detoxification of heavy metals and act as antioxidants, protecting cells against hydroxyl free radicals, including from neuronal damage by excessive NO.^[^ ^27^, ^28^, ^29^ ^]^ It could therefore be that the reduced blood *MT2A* mRNA in children with ASD is implicated in the elevated tyrosine nitration of proteins, following peroxynitrite interaction with proteins, as observed by the Amal team.^[^ ^1^ ^]^

In closing, it is likely that inflammation‐related NO synthesis mediates mitochondrial damage in some brain regions (or specific neuronal or glial cell subtypes) in individuals with ASD. The plausible connection between higher NO levels and mitochondrial dysfunction in ASD deserves further studies, and mitochondrial function measurements should be included in clinical trials examining the potential role of nitric oxide synthase inhibitors in ASD.

## Conflict of Interest

The authors declare no conflict of interest.
